# Population genetic structure of the great star coral, *Montastraea cavernosa,* across the Cuban archipelago with comparisons between microsatellite and SNP markers

**DOI:** 10.1038/s41598-020-72112-5

**Published:** 2020-09-22

**Authors:** Alexis B. Sturm, Ryan J. Eckert, Juliett González Méndez, Patricia González-Díaz, Joshua D. Voss

**Affiliations:** 1 Harbor Branch Oceanographic Institute, Florida Atlantic University, 5600 N US Highway 1, Fort Pierce, FL 34946 USA; 2 Centro Nacional de Áreas Protegidas, Calle 18a, No. 1441, Playa, La Habana, Cuba; 3 Centro de Investigaciones Marinas, Universidad de La Habana, Calle 16, No. 114, Miramar, La Habana, Cuba

**Keywords:** Marine biology, Ecological genetics

## Abstract

Coral reef habitats surrounding Cuba include relatively healthy, well-developed shallow and mesophotic (30–150 m) scleractinian communities at the cross-currents of the Tropical Western Atlantic (TWA). However, Cuba’s coral communities are not immune to the declines observed throughout the TWA, and there is limited information available regarding genetic connectivity, diversity, and structure among these populations. This represents an immense gap in our understanding of coral ecology and population dynamics at both local and regional scales. To address this gap, we evaluated the population genetic structure of the coral *Montastraea cavernosa* across eight reef sites surrounding Cuba. Colonies were genotyped using nine microsatellite markers and > 9,000 single nucleotide polymorphism (SNP) markers generated using the 2bRAD approach to assess fine-scale genetic structure across these sites. Both the microsatellite and SNP analyses identified patterns of genetic differentiation among sample populations. While the microsatellite analyses did not identify significant genetic structure across the seven shallow *M. cavernosa* sampling sites, the SNP analyses revealed significant pairwise population differentiation, suggesting that differentiation is greater between eastern and western sites. This study provides insight into methodological differences between microsatellite and SNP markers including potential trade-offs between marker-specific biases, sample size, sequencing costs, and the ability to resolve subtle patterns of population genetic structure. Furthermore, this study suggests that locations in western Cuba may play important roles in this species’ regional metapopulation dynamics and therefore may merit incorporation into developing international management efforts in addition to the local management the sites receive.

## Introduction

In response to the precipitous declines in coral cover and health on reefs throughout the Tropical Western Atlantic (TWA) and worldwide, greater focus has been placed on quantifying larval connectivity among coral populations that may play critical roles in the persistence and recovery of regional metapopulations^1,2^. Population genetic approaches have been widely implemented to quantify gene flow among coral populations and can be used to infer patterns of connectivity, generating critical information that can be applied to management and conservation strategies^3–7^. Assessments of coral genetic connectivity in the TWA have focused on a variety of coral species with different reproductive mechanisms and life histories^8–10^, examined wide-ranging spatial scales and depth gradients^6,7,11,12^, and employed a variety of molecular markers^13–16^. However, there have been relatively few studies focused on characterizing the connectivity patterns and dynamics in coral genetic structure across the Cuban archipelago or between Cuba and other reefs in the TWA^17–19^.

An assessment of coral connectivity among Cuba’s reef ecosystems is critical as these populations may act as coral refugia within the TWA. Cuba has the largest shelf habitat in the Caribbean, supporting almost 4,000 km of reef^20,21^. The lack of development along Cuba’s coastline and the implementation of low-input agricultural methods has led to comparatively minimal levels of local human impact on Cuba’s coral reefs^22–24^. Based on one dataset spanning from 1988–2007, Cuba has maintained relatively high coral cover, averaging 17.6% and 13.4% along the shallow reef crest and fore reef, respectively^25^. In contrast, when surveyed in 2011, the average coral cover across sites in the nearby Florida Keys was only 2.4%^26^. Study of Cuba’s immense mesophotic coral ecosystems (MCEs, 30–150 m) began with coral community characterization down to 70 m, however deeper and more detailed description of Cuba’s mesophotic communities has only recently been undertaken^27,28^. Reports from a 2017 expedition estimate as much as 80% coral cover and suggest a relatively low incidence of bleaching and disease within these coral communities^29^. The immense habitat area, high coral cover, and relative health of Cuba’s coral communities have contributed to their reputation as an “ecological crown jewel” of the Caribbean Sea^24^.

While Cuban coral reefs are relatively healthy, they are not immune to anthropogenic impacts. Overexploitation of coral reef fishes, land-based pollution, and multiple thermally induced bleaching events have contributed to an estimated average annual decline in coral cover of 1.75%, only slightly lower than the estimated decline for the wider TWA (2.5–2.7%)^2,24,25,30,31^. In response to these declines, Cuba has implemented several strategies to protect its coastal marine ecosystems, including coral reefs. As of 2018, 25% of Cuba’s insular shelf is a designated or proposed marine protected area (MPA), encompassing an estimated 30% of Cuban coral reefs^32^. In addition, government agencies from Cuba and the United States established a “Sister Sanctuary” relationship among the Florida Keys and Flower Garden Banks National Marine Sanctuaries in the U.S. and the physically and ecologically connected Guanahacabibes National Park and Banco de San Antonio Marine Sanctuary in Cuba^33^.

Despite ongoing efforts to manage and conserve these threatened coral communities, there has only been one study focused on characterizing coral genetic structure across sites throughout the Cuban archipelago. Ulmo-Díaz et al*.*^19^ found low levels of genetic diversity among *Orbicella faveolata* sampled throughout Cuba*.* One notable exception was higher levels of diversity observed within the northwestern Los Colorados archipelago and higher levels of genetic differentiation between this site and other reefs. The study presented here aims to investigate patterns of genetic connectivity of a different coral species, *Montastraea cavernosa* (Linnaeus, 1767), across sites surrounding Cuba. *Montastraea cavernosa* is a cosmopolitan^34^, extreme depth-generalist^35,36^, broadcast spawner^37^, and important reef builder^38^. These characteristics, in combination with a number of available molecular resources^10,39,40^, make *M. cavernosa* an excellent candidate species for population genetic studies. Previous investigations have demonstrated varying levels of horizontal and vertical genetic connectivity of *M. cavernosa* across local and regional spatial scales and between shallow and mesophotic depth zones^7,12,14,34,41^.

Population genetic studies have historically implemented multiple classes of molecular markers but microsatellites are one of the most widely used and versatile marker classes in population genetic research^42,43^. Microsatellite markers are highly polymorphic short-sequence repeats that can be easily and inexpensively applied, especially for species where they have already been developed (e.g. *M. cavernosa*). However, the number of microsatellite loci that can be efficiently genotyped for multiple individuals is limited and their high mutation rate may not be reflective of the individual’s genomic dynamics on the whole^44^. These markers also require frequent manual allele calling, which incorporates subjectivity and potential for scoring bias^45^. The implementation of high-throughput sequencing technologies has enabled the development of single nucleotide polymorphism (SNP) markers through restriction site-associated DNA sequencing (RADseq) approaches^46,47^. The 2bRAD approach falls under the umbrella of RADseq techniques, and functions by employing a type IIB restriction endonuclease to cleave short, uniform length DNA sequences upstream and downstream from recognition sites dispersed throughout the genome, oftentimes generating thousands of SNP loci^48^. While the library preparation and sequencing can be more costly and labor-intensive than microsatellites, many studies have found SNPs to be more informative when quantifying population genetic structure, especially in cases where sample size and/or overall levels of genetic differentiation are low^49–51^.

In addition to quantifying coral population genetic structure, characterization of the coral’s associated algal symbiont assemblages is also critical to our understanding of aspects of the species’ ecology. Patterns in algal symbiont community structure may complement patterns of coral host genetic structure or may provide insight into potential drivers of genetic differentiation^52,53^. Detailed characterization of algal symbiont community structure traditionally relies on genetic methods including sequencing of markers like *ITS2*, *psbA*^ncr^, and other candidate genes^54–56^. However, this additional lab work and sequencing can be time-consuming and expensive. With RADseq methods, algal symbiont sequences can be aligned to transcriptomic and genomic references available from the algal symbiont genera^57^. While this approach only generates a proxy for algal symbiont community make-up and is currently limited in its resolution to the genus level, it can provide preliminary insight into algal symbiont communities without additional lab or sequencing efforts. *Montastraea cavernosa* algal symbiont communities are primarily dominated by members of the genus *Cladocopium* (formerly clade C) but these corals may also maintain communities dominated by, or with background levels of, *Durusdinium* (clade D), *Breviolum* (clade B), and *Symbiodinium* (clade A)^10,58–60^.

The primary objective of this study was to characterize patterns of genetic differentiation among sample populations of *M. cavernosa* within Cuba and inform regional management strategies by quantifying connectivity among sites within established or proposed MPAs. The second objective was to compare the efficacy and resolution of both microsatellites and 2bRAD SNP genotyping to holistically describe patterns of coral population connectivity.

## Methods

### Sample collection and processing

**Figure 1:**
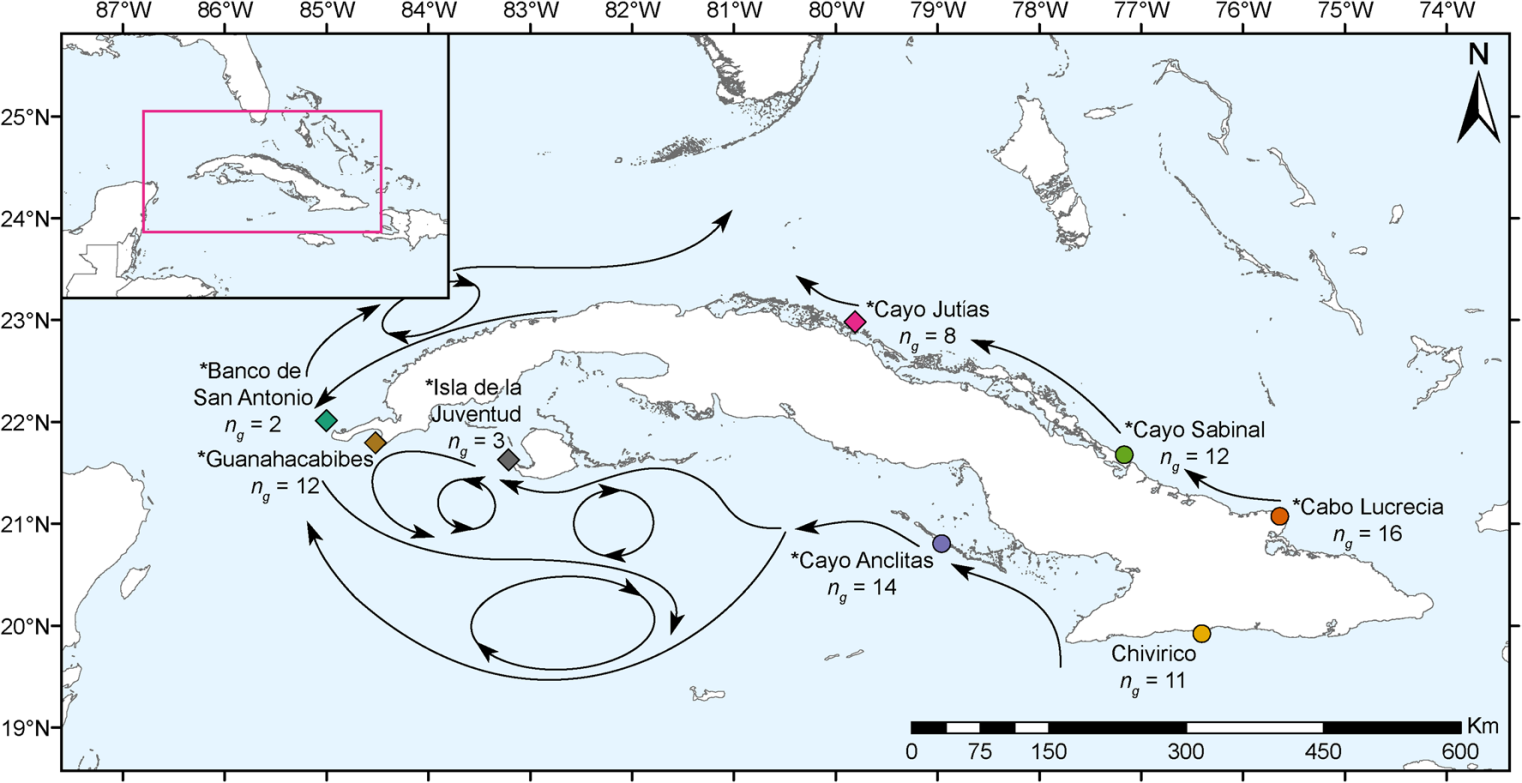
*Montastraea cavernosa* sampling sites throughout Cuba. The number of unique individuals, excluding clones, (*n*_*g*_) included in downstream analyses are indicated for each sample population. Sampling sites indicated by diamonds are considered western populations and sites indicated by circles are eastern populations. Sites with asterisks are located within established or proposed MPAs. Stylized arrows indicate major oceanic currents and circulation patterns surrounding Cuba^18,19,62^. The map was created using ArcGIS v10.4 software developed by ESRI (https://www.esri.com).

*Montastraea cavernosa* samples were collected during May and June 2017 on a joint U.S.-Cuba expedition that circumnavigated the Cuban archipelago to survey and characterize the geomorphology, biodiversity, and ecological health status of Cuba’s shallow and mesophotic coral ecosystems^61^. Ninety-four shallow *M. cavernosa* colonies were sampled from depths between 1–5 m by snorkelers using a hammer and masonry chisel. An additional two mesophotic samples were collected at 41 and 75 m, respectively, by the *Mohawk* remotely operated vehicle (ROV). Unfortunately, the flattened colony morphology of this species at mesophotic depths, in combination with limitations in the strength and dexterity of the ROV’s sampling apparatus, restricted the ability to effectively collect mesophotic tissue samples. A total of 96 *M. cavernosa* samples (~ 5 cm^2^ each) were collected across eight sampling sites surrounding the island (Fig. 1, Table 1).

**Table 1:** *Montastraea cavernosa* samples across eight sites in Cuba including field samples collected (*n*_*c*_= 96), number of samples used in downstream analyses (*n*_*a*_ = 80)*,* and number of unique multi-locus genotypes identified after removal of clones (*n*_*g*_ = 78).

Sampling site	Sampling date	Depth range (m)	*n*_*c*_	*n*_*a*_	*n*_*g*_	Latitude	Longitude
Banco de San Antonio	21 May 2017	41; 75	2	2	2	22.01502	− 84.99913
Guanahacabibes	23 May 2017	3–5	16	13	12	21.79668	− 84.51667
Isla de la Juventud	25 May 2017	3–5	3	3	3	21.63063	− 83.21420
Cayo Anclitas	1 June 2017	3–5	15	14	14	20.80792	− 78.95550
Chivirico	3 June 2017	3–5	15	12	11	19.92550	− 76.40367
Cabo Lucrecia	6 June 2017	3–5	17	16	16	21.07500	− 75.63833
Cayo Sabinal	7 June 2017	1–4	15	12	12	21.67833	− 77.16667
Cayo Jutías	9 June 2017	1–2	13	8	8	22.98263	− 79.80660

GPS coordinates are in decimal degrees (WGS84).

All samples were preserved in TRIzol reagent and maintained at -20 °C before transport back to Florida Atlantic University (FAU) Harbor Branch Oceanographic Institute where they were stored at -80 °C prior to DNA extraction. Total genomic DNA was extracted from these samples using a modified CTAB extraction protocol as described in Eckert et al*.*^12^, then purified and concentrated using a Zymo Research DNA Clean & Concentrator-5 kit following manufacturer’s protocols. The quality and concentration of samples were measured on a NanoDrop 2000 spectrophotometer (Thermo Fisher Scientific) and a Qubit 2.0 fluorometer (Invitrogen). Samples were subsequently diluted to equal concentrations before microsatellite amplification and 2bRAD library preparation.

### Microsatellite amplification and genotyping

Samples were genotyped using nine previously developed microsatellite loci^10^, amplified in triplex with self-labeled fluorescent primers using the QIAGEN Type-It Microsatellite PCR kit following modified methods outlined in Eckert et al*.*^12^. Successful amplification was verified through visualization on a 2% agarose gel before amplicons were diluted in deionized water and measured using an Applied Biosystems ABI 3130xl genetic analyzer with ROX-500 size standard. Generated electropherograms were used to call alleles in GeneMapper v3.7 following protocols as described by Studivan and Voss^7^. Samples with loci that did not amplify successfully or were of otherwise poor quality were re-amplified to produce the most complete dataset possible. In addition, a random subset of samples was re-amplified and re-analyzed to ensure consistency across analyzer runs and allele calls. Samples were amplified and run an average of 1.3 times and only samples genotyped at six or more loci were used in further analyses.

### SNP library preparation and genotyping

2bRAD SNP libraries were prepared following Wang et al*.*^48^ including modifications described in the protocol’s GitHub repository (https://github.com/z0on/2bRAD_denovo). Briefly, the protocol’s five main steps include: (1) restriction digest of 200 ng of total genomic DNA with BcgI endonuclease; (2) ligation of DNA fragments with indexed adaptors; (3) pooling of indexed ligations; (4) PCR amplification of pooled ligations; (5) and size-selection and purification of ~ 180 bp fragments. Library preparation time and cost were significantly reduced through the use of 12 uniquely indexed 3′ adaptors, allowing 12 sample ligations to be pooled prior to amplification. Fully degenerate 5′ adaptors were also included to allow filtering of PCR duplicates in downstream analyses. Triplicate 2bRAD libraries were prepared for three coral samples as described in Manzello et al*.*^57^. These triplicate libraries were used downstream as a sequencing quality check and to identify natural clones. Pooled libraries were further pooled in equimolar amounts based on qPCR relative quantification. The final pooled library underwent automated size selection with Pippin Prep (Sage Science) before conducting 100-bp single-end sequencing on an Illumina NovaSeq S1 flow cell at the University of Texas at Austin’s Genome Sequencing and Analysis Facility.

Reads were initially demultiplexed by the sequencing facility using PCR and TruSeq indices and were further demultiplexed by the in-line 3′ adaptor indices, deduplicated, and trimmed using custom Perl scripts (https://github.com/z0on/2bRAD_denovo). The sequences were further quality-filtered using the fastq_quality_filter in the FASTX-Toolkit v0.0.14^63^ only retaining reads with at least 90% of bases with Phred quality scores ≥ 20. Trimmed and quality-filtered reads were aligned to the *M. cavernosa* genome (version July 2018, https://matzlab.weebly.com/data--code.html)^40^; concatenated with available algal symbiont transcriptomes for the Symbiodiniaceae genera *Symbiodinium*^64^, *Breviolum* (https://sites.bu.edu/davieslab/data-code/), *Cladocopium*^65^, and *Durusdinium*^66^ using the sequence aligner *Bowtie2* v2.3.5^67^. Reads that mapped with high levels of uniqueness to each of the algal symbiont transcriptomes (mapping quality ≥ 40) were counted using custom Perl scripts as described by Manzello et al*.*^57^ and used as a proxy for the relative abundance of these four algal symbiont genera associated with each sample. Only reads that aligned to the *M. cavernosa* genome were included in downstream analyses of *M. cavernosa* population genetic structure.

Genotype likelihoods were calculated using the program ANGSD v0.921, which incorporates probabilistic uncertainty into the SNP genotyping process, making it a more robust method to use for variable, low, or medium sequencing depth coverage^68^. ANGSD was run with the following filters: minimum mapping quality scores of 20, minimum base quality scores of 25, *p*-value of 10^–5^ that a SNP is true, at least 75% of non-missing genotypes across samples, minimum *p*-value for deviation from Hardy–Weinberg equilibrium of 0.05, minimum *p*-value for strand bias of 0.05, minimum allele frequency of 0.05, and a filter that removed any tri-allelic SNPs.

### Microsatellite population genetic structure analyses

Of the 96 samples collected, 95 were successfully amplified across ≥ 6 microsatellite loci. However, only a trimmed dataset of 80 samples was used in the downstream analysis to maintain a consistent sample set between the microsatellite and 2bRAD analyses. Observed and expected heterozygosity, fixation index (*F*_ST_), and Nei’s genetic distance were calculated in GenAlEx v6.503^69^. Analysis of Molecular Variance (AMOVA) was calculated in GenAlEx using *F*_ST_ to assess population differentiation^70^ (9,999 model and population permutations). Pairwise *F*_ST_ value comparisons between each site were also generated in GenAlEx and associated false discovery rate (FDR)-corrected *p-*values were calculated^71^ (9,999 permutations). Population differentiation was visualized using Principal Coordinates Analysis (PCoA) based on Nei’s genetic distance.

The R package *poppr* v2.8.3 was used to calculate Prevosti’s absolute genetic distances among samples to construct a dendrogram to identify genotypic clustering of samples^72–74^. A Principal Components Analysis (PCA) was conducted on the microsatellite genotypes using the R package *adegenet* v2.1.1^75^. A Mantel test (isolation-by-distance) was used to measure the correlation between Nei’s genetic distance and geographic distance among sampling populations’ geographic coordinates using the function *mantel.randtest* from the R package *adegenet*^75,76^ (999 permutations).

The Bayesian model-based clustering program STRUCTURE v2.3.4 was run via the R package *ParallelStructure* v1.0 on FAU’s high-performance computing cluster to assess population structure^77,78^ (10 replicate simulations, 10^3^ burn-in iterations, and 10^6^ Markov Chain-Monte Carlo replicates). Models were run for genetic cluster values *K* = 1–11, or the number of sampling sites + 3 to identify any possibility of cryptic genetic clusters within sampling populations. The LOCPRIOR option was selected to include information on the sample location as a model input. The web-based program STRUCTURE HARVESTER v0.6.94 was used to generate model likelihoods for different values of *K*^79^. STRUCTURE plots were generated in R.

### SNP population genetic structure analyses

ANGSD was used to compute an identity-by-state (IBS) matrix to observe sample clustering and to identify genetic clones. The IBS approach calculates the proportion of times that two randomly selected reads that contain a certain SNP locus are the same or different between two individuals. This measurement of genetic distance is particularly robust to variation in sequencing coverage among samples^57^. The resulting pairwise IBS matrix was used to generate a cluster dendrogram using the function *hclust* in R. This dendrogram was used to identify naturally occurring clones that exhibited similar levels of genetic similarity to one another as the technical triplicate groups. The dataset was then edited to remove replicates and natural clones.

ANGSD was re-run with the same filter parameters on the dataset with clones removed. The newly generated IBS matrix was used to produce a new cluster dendrogram. A PCA and AMOVA (999 permutations) were conducted on the genotype likelihoods produced by ANGSD using the R packages *poppr* and *adegenet.* Observed and expected heterozygosities were calculated from ANGSD outputs using the *Stacks* v2.3 populations program^80^. Nei’s genetic distance and pairwise *F*_ST_ values were calculated using the R package *StAMPP* v1.5.1^81^ (99 permutations). A PCoA of population-level Nei’s genetic distance was conducted using the *cmdscale* function in the R package *vegan* v2.5.6^82^. A Mantel test was used to measure the correlation between Nei’s genetic distance and geographic distance among sampling populations using the function *mantel.randtest* from the R package *adegenet* (999 permutations).

Population structure for models of cluster values *K* = 1–11 were assessed with ADMIXTURE v1.3.0^83^, which uses the same statistical model as STRUCTURE to estimate an individual’s ancestral membership but applies an algorithm optimized for large multi-locus SNP datasets. Cross-validation error values calculated for each model identified the most likely values of *K.* The program NGSadmix v32 was employed to conduct ADMIXTURE analysis on genotypic likelihoods in lieu of hard-called genotypes^84^. NGSadmix plots were generated in R.

Outlier SNP loci putatively undergoing selection were identified based on locus-population-specific differences in *F*_ST_ values using the program BAYESCAN v2.1^85^ (50,000 burn-in, 5,000 iterations) and plotted with R (https://github.com/z0on/2bRAD_denovo). The locations of these outlier SNP loci were compared against annotated gene regions (extended by ± 2 kb) of the *M. cavernosa* genome to identify any putative functional selection effect.

## Results

### Data summary and identification of genetic clones

**Figure 2:**
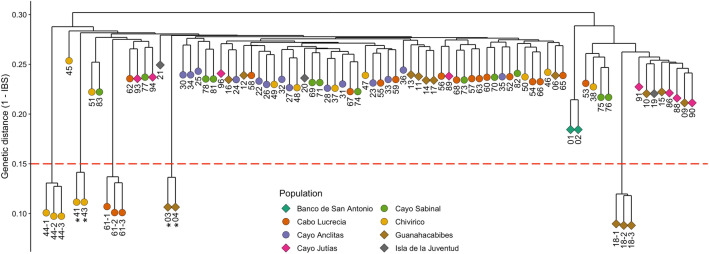
Hierarchical cluster dendrogram based on pairwise identity-by-state (IBS) values from SNP data for all samples. Samples from western sites are indicated by diamonds and samples from eastern sites are indicated by circles. Three sets of technical triplicates for samples 18, 44, and 61 are shown and indicated by the sample number and a hyphen. IBS levels calculated for technical replicates were used to determine a threshold by which to identify natural clones that fall below the dashed red line. Two pairs of natural clones, indicated by asterisks, were also identified as natural clones in the microsatellite analysis based on multi-locus genotypes.

In the microsatellite dataset, all of the 80 samples were genotyped across six or more loci and 73 of the samples (91%) were genotyped across all nine microsatellite loci. Two pairs of samples were identified as genetic clones based on their identical multi-locus genotypes. These clones were samples collected from the Guanahacabibes and Chivirico sites. In situ collection photos were reviewed to verify that these samples were true genetic clones and not accidental re-samples of the same colony. From the 2bRAD sequencing, over 275 million reads were obtained with an average of 3.21 million reads per sample library. Following the removal of PCR duplicates and quality-filtering, there were over 127 million reads retained, averaging 1.48 million reads per sample. Hierarchical clustering based on IBS generated from the SNP dataset discerned the same two pairs of natural clones also identified in the microsatellite dataset (Fig. 2). After the removal of the genetic clones and technical replicates, there were a total of 78 individual samples. ANGSD was re-run on the clones removed SNP dataset which generated a total of 9,720 SNP loci identified across a minimum of 75% of the samples.

### Genetic differentiation and population structure

**Figure 3:**
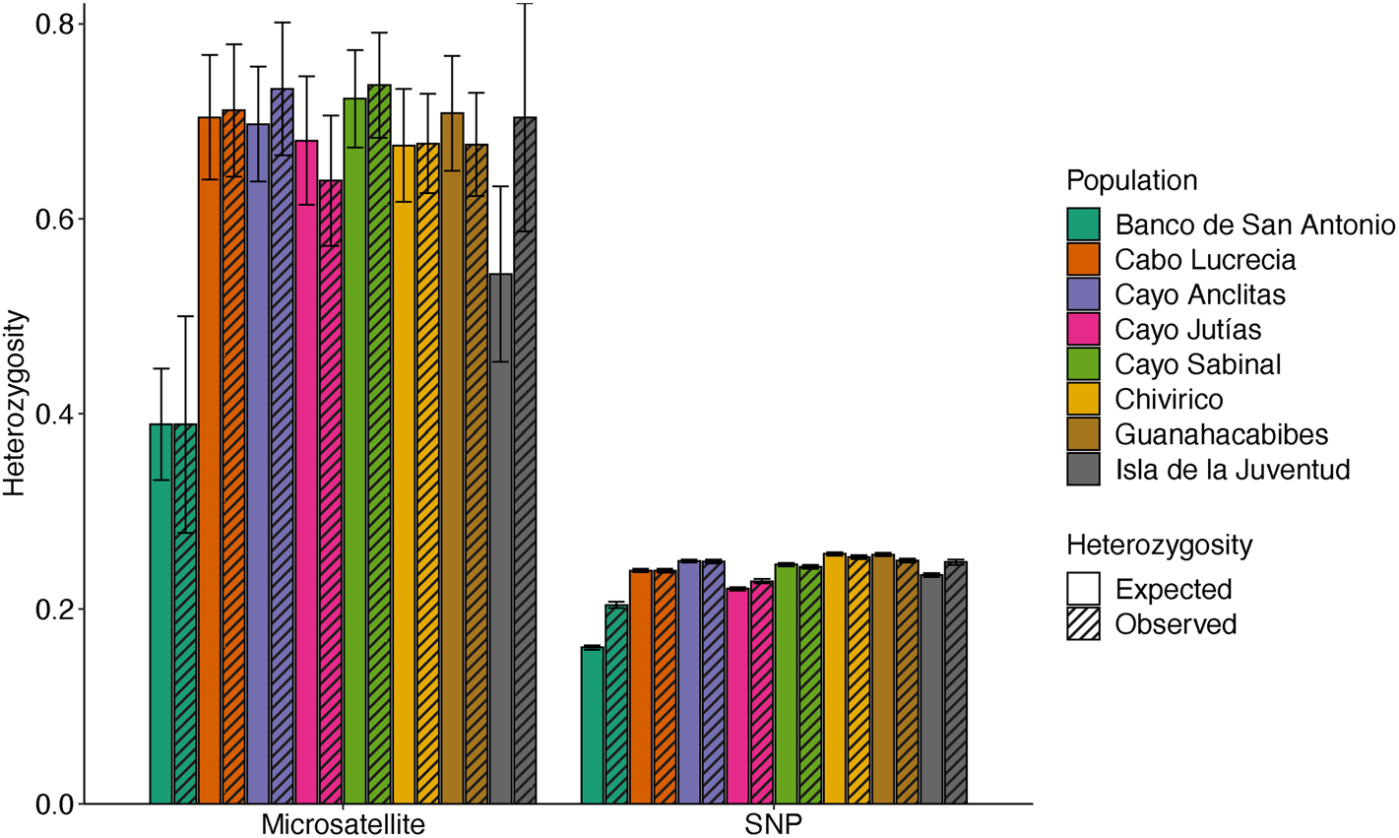
Mean expected (*H*_e_) and observed (*H*_o_) heterozygosity (± SE) calculated for each sampling population and generated for both the microsatellite and SNP datasets.

Mean observed and expected heterozygosity calculated from the microsatellite dataset were higher across all populations than the SNP dataset (Fig. 3, Supplementary Table S1). Heterozygosity values varied more widely across populations in the microsatellite dataset and were especially lower in the Banco de San Antonio samples compared to other populations (*H*_o_ = 0.389 ± 0.111, *H*_e_ = 0.389 ± 0.057). Heterozygosity values based on the SNP dataset were more consistent across populations (*H*_o_ = 0.204–0.254, *H*_e_ = 0.161–0.256), however, the Banco de San Antonio average heterozygosity values were still lower than the other populations, possibly due to low sample size.

**Figure 4:**
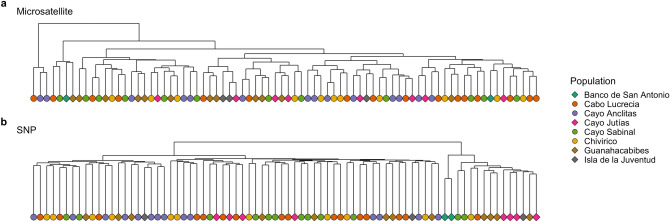
Hierarchical clustering of genotyped corals by genetic distance using (**a**) Prevosti’s absolute genetic distance for the microsatellite dataset and (**b**) identity-by-state distance for the SNP dataset. Samples from western sites are indicated by diamonds and samples from eastern sites are indicated by circles.

The hierarchical cluster dendrogram generated from the microsatellite dataset based on Prevosti’s genetic distance did not produce discernible clustering patterns among samples (Fig. 4a). In contrast, the cluster dendrogram based on the IBS matrix generated from the SNP dataset clustered the samples into two main groups, one group with the majority of samples (63) and an out-grouping of only 15 samples which was further sub-divided into the two mesophotic samples from Banco de San Antonio and a cluster of an additional 13 shallow samples primarily from the western sampling sites (Fig. 4b).

**Figure 5:**
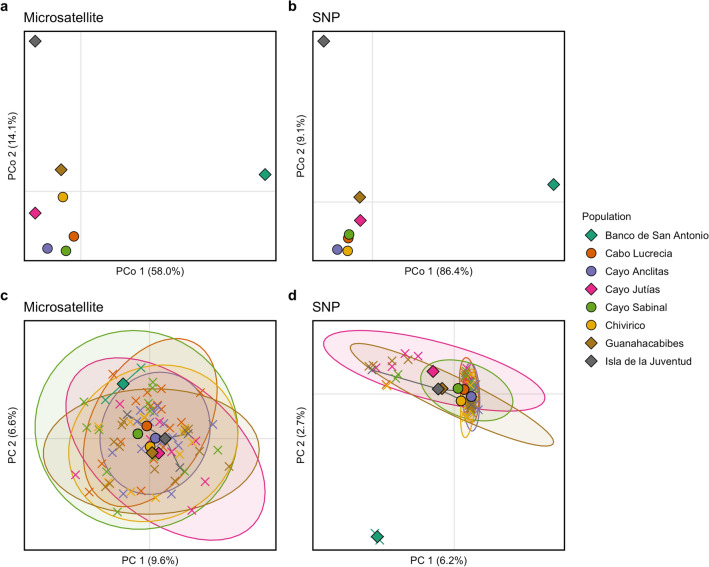
Principal coordinates analysis based on Nei’s genetic distance matrix of pairwise population comparisons for (**a**) microsatellites and (**b**) SNPs. Principal components analysis visualizing clustering of individual samples indicated by ‘X’s and population centroids are indicated by diamonds for western sites and circles for eastern sites for (**c**) microsatellites and (**d**) SNPs. Percent variation explained by each axis is indicated.

AMOVA performed for both the microsatellite and SNP datasets indicated low but significant levels of genetic differentiation among populations (microsatellite: 0.96%, SS = 28.03, *df*_7,155_, *p* = 0.03; SNP: 1.88%, SS = 12,345.90, *df*_7,155_, *p* = 0.0001). PCoA visualization of Nei’s genetic distance matrices generated similar plots for both the microsatellite and SNP datasets, with clustering of the Guanahacabibes, Cayo Jutías, Cayo Sabinal, Cabo Lucrecia, Cayo Anclitas, and Chivirico populations while the Banco de San Antonio and Isla de la Juventud populations were relatively distanced from the main cluster (Fig. 5a, b).

PCA on the microsatellite dataset demonstrated clustering of the shallow sites with some separation of the mesophotic Banco de San Antonio samples, however, there were no distinctive patterns among the shallow samples (Fig. 5c). In contrast, the SNP dataset demonstrated similar patterns of clustering as the IBS cluster dendrogram with a core cluster consisting of the majority of samples and two out-groupings, the two samples from the mesophotic site, Banco de San Antonio and the 13 samples predominantly from western populations (Fig. 5d). Western sites were split from eastern sites along PC1. Within the western and eastern clusters, further splits between northern and southern sites occurred along PC2, with northwestern Cayo Jutías separated from southwestern Guanahacabibes and Isla de la Juventud and northeastern Cayo Sabinal and Cabo Lucrecia separated from southeastern Cayo Anclitas and Chivirico. Bayesian information criterion (BIC) analysis selected *K* = 2 as the optimal number of clusters to explain PCA variation based on the SNP dataset (Supplementary Fig. S1d).

**Figure 6:**
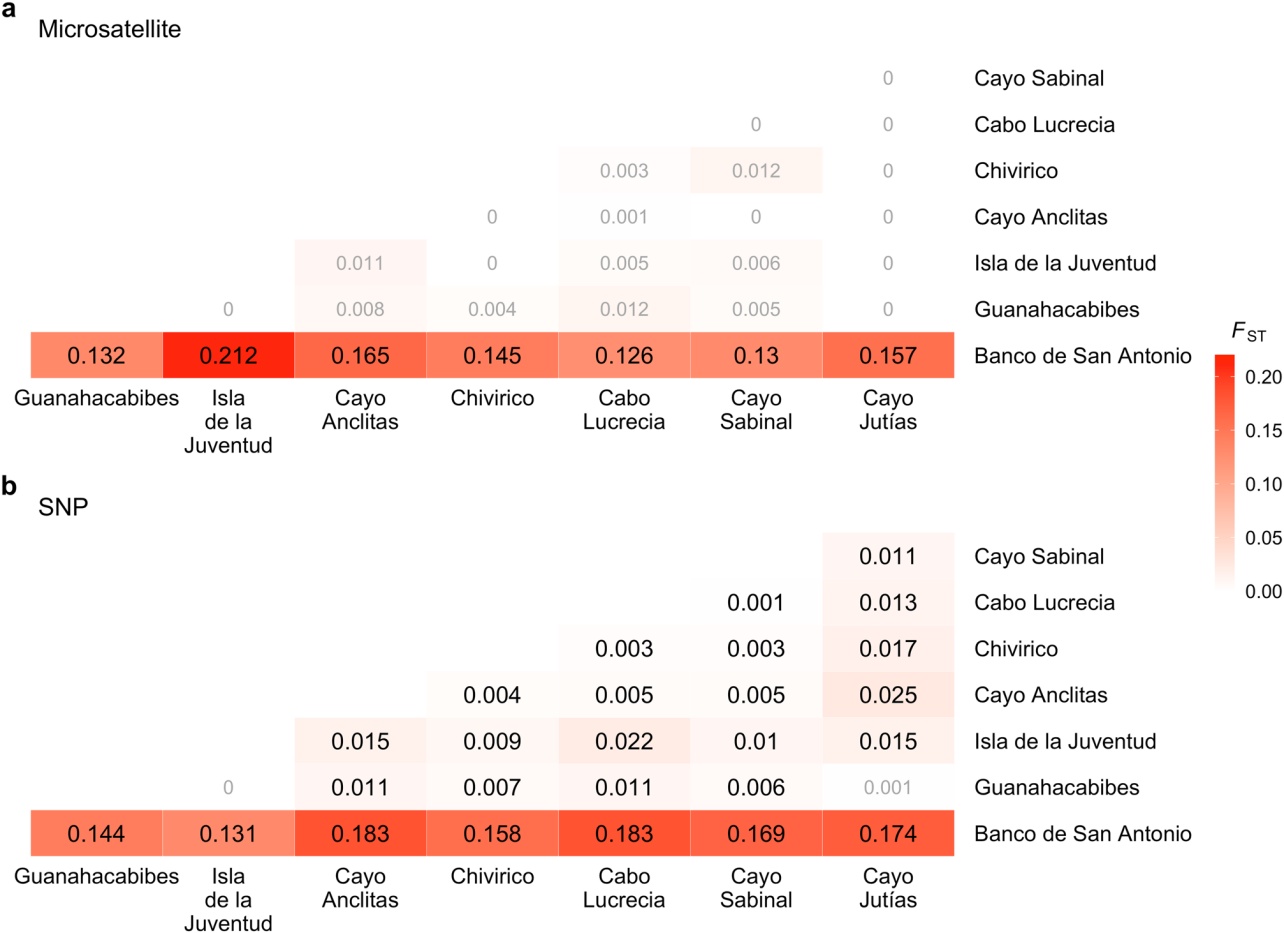
Heat map representations of pairwise population differentiation as estimated by fixation index (*F*_ST_) for (**a**) microsatellite and (**b**) SNP datasets. Values within cells are estimated *F*_ST_ with increasing intensity of the color red corresponding to increasing *F*_ST_ values. Bolded *F*_ST_ values denote significant differentiation between populations (post FDR-correction, *p* < 0.05).

Pairwise population *F*_ST_ values varied widely among comparisons and between the microsatellite and SNP datasets (Fig. 6). Similar to the patterns indicated by the ordination plots, the highest pairwise *F*_ST_ values in both datasets were between the Banco de San Antonio sample population and all other populations. In the microsatellite dataset, these were the only significant pairwise *F*_ST_ comparisons identified (FDR-corrected *p* < 0.05), with all shallow to shallow pairwise population *F*_ST_ comparisons found to be non-significant (Fig. 6a). Pairwise *F*_ST_ values from the SNP dataset tended to be slightly higher than their microsatellite counterpart for some, but not all, of the comparisons. However, the majority of pairwise *F*_ST_ comparisons, including shallow to shallow population comparisons, were significant (FDR-corrected *p* < 0.05, Fig. 6b).

Mantel tests identified significant positive correlations between genetic and geographic distances among the sites for both datasets (microsatellite: *r* = 0.241, *p* = 0.013, SNP: *r* = 0.235, *p* = 0.016, Supplementary Fig. S2, suggesting that *M. cavernosa* samples from more geographically distant sites display higher levels of genetic differentiation.

**Figure 7:**
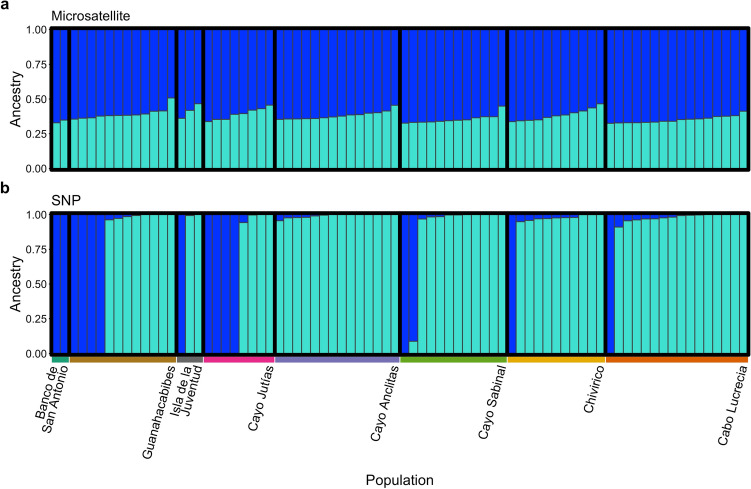
Genetic structure plots demonstrating estimated membership of each *Montastraea cavernosa* sample to each of the two proposed genetic clusters (*K* = 2 model). Genetic structure analysis conducted using the program STRUCTURE for (**a**) microsatellite analysis and the program NGSadmix for (**b**) SNP analysis.

Population structure analysis of the microsatellite dataset using the program STRUCTURE estimated *K* = 2 as the most likely number of genetic clusters based on the Evanno method^86^ (Supplementary Fig. S1b). Notably, the Evanno method cannot consider *K* = 1 in its evaluation of model likelihood as it is an ad hoc statistic based on the rate of change in the log probability of data between successive *K* values. This result was in disagreement with *K* selection based on the log model likelihood values (L(*K*)), which suggested that *K* = 1 was the most likely number of genetic clusters (Supplementary Fig. S1a). A STRUCTURE bar plot was created for *K* = 2 but did not identify any patterns in genetic divergence across the sampling populations (Fig. 7a).

Population structure analysis conducted on the SNP dataset using the programs NGSAdmix and ADMIXTURE were in agreement with the microsatellite STRUCTURE analysis suggesting that *K* = 1 was the most likely number of genetic clusters based on the lowest model cross-validation error (Supplementary Fig. S1c). Error for the *K* = 2 model was only 0.3% higher than the *K* = 1 model and was the most likely number of clusters based on BIC analysis. Therefore, NGSadmix was used to generate a *K* = 2 bar plot (Fig. 7b). The majority of samples were almost completely dominated by the turquoise cluster, while the same 15 samples identified in the PCA and the IBS cluster dendrogram were dominated by the dark blue cluster and were predominantly individuals from western sites.

### Outlier loci

Only 11 loci (0.11% of all genotyped SNP loci) were identified as candidate SNPs undergoing selection by BAYESCAN based on their significant locus-specific *F*_ST_ values (FDR-corrected *p* < 0.1). All of these loci had positive alpha values suggesting that they are undergoing diversifying selection^85^. Four of these loci were identified within ± 2 kb of an *M. cavernosa* annotated gene region with assigned gene ontologies (version July 2018, https://matzlab.weebly.com/data--code.html)^40^ functioning primarily in inorganic ion transport, protein modification and turnover, and signal transduction (Table 2).

**Table 2:** List of candidate SNP loci putatively undergoing selection within annotated *Montastraea cavernosa* gene regions, identified by BAYESCAN based on their outlier *F*_ST_ values (FDR-corrected *p* < 0.1).

Locus Number	Chromosome	Location	Gene Alias	eggNOG Annotation	COG Categories
986	Sc0000029	237,612	Mcavernosa03716	Polycystic kidney disease 1-like 2	Signal transduction mechanisms; Inorganic ion transport and metabolism
987	Sc0000029	237,623	Mcavernosa03716	Polycystic kidney disease 1-like 2	Signal transduction mechanisms; Inorganic ion transport and metabolism
1,397	Sc0000046	322,120	Mcavernosa24377	Peptidylglycine alpha-amidating monooxygenase	Post-translational modification, protein turnover, and chaperones
9,718	xpSc0007425	1,843	Mcavernosa35228	Neuronal cell adhesion molecule	Signal transduction mechanisms

The locus number and location of each outlier SNP locus, associated gene alias, functional annotation, and associated clusters of orthologous groups (COG) categories are listed.

### Algal symbiont characterization

**Figure 8:**
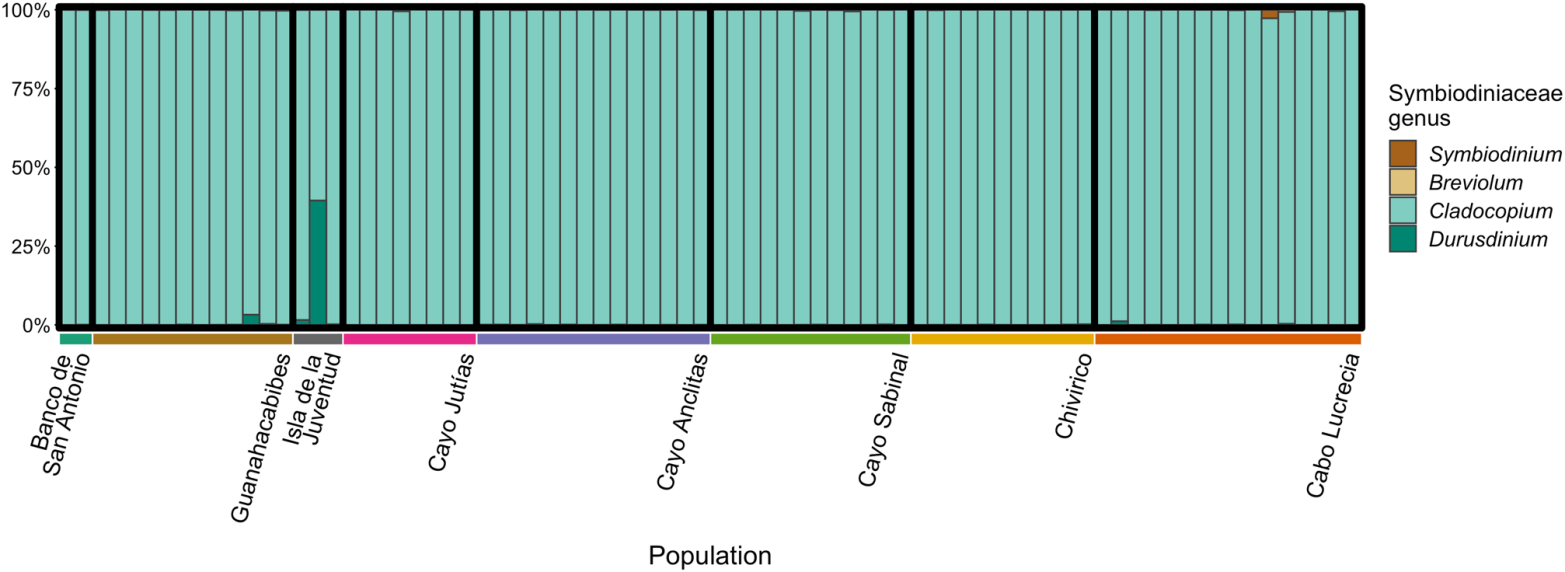
Bar plot representing a proxy for algal symbionts of each coral sample based on highly unique mapped 2bRAD reads (mapping quality ≥ 40) to transcriptomes of four different genera of algal symbionts, *Symbiodinium, Breviolum, Cladocopium,* and *Durusdinium* (formerly Clades A–D, respectively).

The majority of reads that aligned to the algal symbiont transcriptomes aligned to the *Cladocopium* transcriptome. Only a small portion of reads aligned to *Symbiodinium*, *Breviolum*, or *Durusdinium* transcriptomes, suggesting that *Cladocopium* is the dominant genus of algal symbionts hosted by *M. cavernosa* in Cuba (Fig. 8). The mesophotic samples from the Banco de San Antonio site were also dominated by *Cladocopium* (99.9% and 99.8% of their mapped algal symbiont reads) and did not show large differences in proxy symbiont associations from the majority of the shallow populations across the other seven sites.

## Discussion

### Genetic differentiation between eastern and western Cuban M. cavernosa populations

*Montastraea cavernosa* from eight locations surrounding the Cuban archipelago exhibited significant levels of genetic differentiation among sites. PCoA, PCA, isolation by distance analysis, and pairwise *F*_ST_ values generated from the SNP dataset generally support a pattern of higher differentiation between western and eastern sites (Fig. 1). A similar pattern was observed among three *Orbicella annularis* sample populations in Cuba by Foster et al*.*^17^; the northeastern and southeastern sites were more highly differentiated from the northwestern site than they were from one another. Similarly, significant genetic differentiation between western and eastern populations have been observed for two species of Penaeid shrimp collected from sites along the southern Cuban coast^19^.

Much of the east–west differentiation pattern observed in this study is driven by 15 samples identified in the SNP dataset that clustered together and away from the other samples in the IBS cluster dendrogram and PCA (Figs. 4b, 5d). These same 15 samples were also dominated by the dark blue genetic cluster in the NGSadmix analysis (Fig. 7b). Eleven of these 15 samples were from the western sites, which are likely influenced by the powerful, regional currents that make up the Gulf Stream current system^87^ (Fig. 1). This current system first travels northward as the Yucatan Current alongside the Mesoamerican Reef, reaching mean maximum speeds along its western edge of 110 cm s^-1^ and driving a southernly countercurrent along the western tip of Cuba reaching speeds of > 20 cm s^-1 ^^88^. The Yucatan Current then enters the Gulf of Mexico through the Yucatan Channel and becomes the Loop Current^87^. The current bends eastward and exits through the Straits of Florida as the Florida Current traveling along Cuba’s northwestern coast where it reaches maximum annual mean velocities of 115 cm s^-1 ^^88^. In addition, mesoscale anticyclonic eddies are shed from the Florida Current where they interact with the western and northwestern coast of Cuba^89^. One possible hypothesis is that the 15 samples dominated by the dark blue cluster are members of a “regional” genetic cluster and may be more closely related to individuals from physically connected reef populations up- or downstream. The turquoise genetic cluster, the dominant cluster in the majority of the samples, may be members of a “local” genetic cluster within Cuba. While this local genetic cluster was found across the island, it more commonly dominated the eastern sites. These sites experience considerably weaker (maximum average surface current speeds < 15 cm s^-1^), more ephemeral, and localized hydrodynamic regimes compared to the western sites which may result in decreased levels of immigration and higher levels of local larval retention^88,90,91^. To evaluate the “local” and “regional” cluster hypothesis, future studies should assess the levels of genetic connectivity of each of these clusters to other population outgroups across the TWA.

While the duration of the obligatory pre-competency period and pelagic larval duration of *M. cavernosa* is presently unknown in situ, laboratory studies of larval survivorship and settlement rates have suggested that larvae become competent to settle after ~ 4 days and have an average larval life expectancy of ~ 15 days^92,93^. The Gulf Stream current system reaches its peak annual transport levels in late July, coinciding with *M. cavernosa* spawning events that occur following the full moons between July and September^37,88^. Based on the velocities of these current systems and estimated maximum pelagic larval duration of *M. cavernosa*, populations from western and northwestern Cuba potentially exhibit some level of larval connectivity with other reef populations in the region, including the Mesoamerican reef, Flower Garden Banks, and Florida Keys. This hypothesis is consistent with conclusions made by a number of biophysical modeling studies. Studies modeling connectivity of snapper species throughout Cuba found higher levels of larval retention in the north-central and southeastern regions and relatively higher levels of connectivity and export in northwestern and southwestern sites^94^. Additionally, another study assessed regional connectivity networks of multiple reef-associated species including the scleractinian corals *O. annularis* and *Porites astreoides*^95^*. O. annularis* has similar life history characteristics to *M. cavernosa,* it is a broadcast spawner, releasing gametes in the late summer with an estimated maximum pelagic larval duration of 30 days^37,95^. In contrast, *P. astreoides* is a brooding species that releases larvae throughout the spring and summer but with a much shorter estimated maximum pelagic larval duration, 7 days^95,96^. Despite these varied life histories, the biophysical model identified populations in western Cuba and north-central Cuba as highly connected source/sink regions for both of these species, while southern Cuba tended to experience higher self-regional recruitment and lower connectivity to upstream sites^95^.

A regional population genetic study of *M. cavernosa* found high levels of genetic connectivity between mesophotic populations in Belize and shallow populations in the Dry Tortugas despite a separation of more than 1,000 km^7^. Sites in western Cuba may serve as a “stepping-stone” for connectivity between these two populations. Therefore, incorporation of *M. cavernosa* samples from Cuba into regional connectivity studies combining high-resolution population genetics and biophysical modeling approaches may offer greater insight into regional connectivity patterns and context for the observed patterns of genetic differentiation within Cuba.

### High levels of genetic differentiation between mesophotic Banco de San Antonio and shallow sample populations

Notably, the highest levels of observed genetic differentiation were between Banco de San Antonio, the only mesophotic population, and all other shallow populations. Pairwise *F*_ST_ values, PCoAs, and PCAs generated from both the microsatellite and SNP dataset demonstrated comparatively high levels of differentiation between Banco de San Antonio and the other sample populations. Given the low sample size from a single mesophotic site, this study cannot make generalized observations on vertical connectivity between shallow and mesophotic coral populations in Cuba. However, these results suggest that further collection of mesophotic coral samples, especially with paired shallow site sample collection to minimize horizontal distance, is critical to fully characterize the connectivity dynamics of this species across Cuba’s extensive shallow and mesophotic habitat. Patterns of connectivity between shallow and mesophotic *M. cavernosa* have been variable in other locations across the TWA. *Montastraea cavernosa* from the Flower Garden Banks exhibit relatively high levels of vertical connectivity, in contrast to populations on the Belize Barrier Reef which exhibit a strong genetic break between shallow and upper mesophotic depth zones^7,12^. *Montastraea cavernosa* populations in Florida show varying levels of genetic differentiation across a large geographic range with lower mesophotic populations at Pulley Ridge exhibiting high levels of differentiation from downstream, shallow populations in the northern Florida Keys, and upper-mesophotic populations in the Dry Tortugas functioning as a genetic intermediate between the two^41^.

### SNPs potentially under selection

Outlier SNPs identified as candidate loci undergoing selection by BAYESCAN represented a minority of the SNP loci identified by ANGSD (0.11% of all genotyped SNP loci). The prevalence of outlier SNP loci in this study is much lower than what has been observed in other coral population genetic studies based on RADseq SNP approaches. For example, 3.1% of the identified SNP loci across *Acropora cervicornis* populations in the Florida Reef Tract were considered outlier loci^97^. Similarly, outlier SNP loci accounted for 3.3% of the total SNPs identified across populations of *Acropora palmata* throughout the Caribbean^98^. While species-level, sequencing, or methodological differences may be driving this distinction in the observed number of SNP loci, these relatively few outlier loci suggest that selective forces on the studied populations are minimal compared to neutral drivers of genetic differentiation among *M. cavernosa* populations in Cuba^99^.

Of the identified outlier loci, four are within or near annotated gene regions that may have functional implications for corals (Table 2). Two outlier SNP loci are located in a protein-encoding gene region involved in calcium ion transporters and is implicated in calcification and skeletal organic matrix formation in coral transcriptomic and proteomic studies^100,101^. Although, given that these two loci were close to one another and identified in the same gene region, they may be misattributed structural variants. A third outlier SNP is located near a gene region involved in responses to oxidative stress in *O. faveolata* larvae exposed to UV radiation^102^. The last outlier SNP was identified in a region annotated as a gene family involved in signal transduction mechanisms that may play roles in coral fertilization and/or in maintaining coral-algal symbiosis^103,104^. As this methodology only identifies candidate outlier loci potentially undergoing selection, further transcriptomic and physiological studies would be needed to fully understand any putative functional effects.

### *Cladocopium* dominated Symbiodiniaceae associations

The dominance of reads aligning to the *Cladocopium* genus from the Cuban *M. cavernosa* samples in this study is consistent with numerous reports that *M. cavernosa* maintains symbioses with this algal genus across site, depth, and time^10,53,105–107^. However, *Cladocopium* is a species-rich genus encompassing genetically, physiologically, and ecologically diverse members^108,109^. Presently, amplicon sequencing of markers such as *ITS2* is needed to assess algal symbiont assemblages to the sub-genus level^54^. Hypothetically, if more algal symbiont genomes are sequenced and published, RADseq methods could be used to effectively conduct simultaneous SNP mining for both coral host and algal symbionts. Nevertheless, the approach used in this study and others demonstrates the ability to use coral holobiont sequence data generated with SNP approaches to broadly characterize *in hospite* algal symbionts^57^.

### Comparisons of microsatellite and 2bRAD SNP approaches

Across multiple population genetic analyses, including hierarchical clustering of samples, PCoA, PCA, AMOVA, pairwise *F*_ST_ calculations, and STRUCTURE/ADMIXTURE analyses, the dataset based on > 9,000 SNPs more clearly and consistently identified ecologically relevant patterns of genetic differentiation in Cuban *M. cavernosa* populations than the dataset based on nine microsatellite markers. In addition, genetic parameters such as heterozygosity were less biased by a low sample size per population in the SNP dataset than the microsatellite dataset. While both approaches were able to identify signatures of significant differentiation between mesophotic and shallow samples, the microsatellite dataset was unable to identify significant genetic differentiation among the seven shallow populations. Notably, assessing the microsatellite dataset alone would suggest that shallow *M. cavernosa* across Cuba are highly admixed and well-connected (Fig. 6a). These results concur with numerous microsatellite versus RADseq/SNP comparative assessments of population genetic structure and individual level genetic diversity in a variety of biological systems^50,51,110,111^. These studies have routinely demonstrated that SNP approaches outperform microsatellites in the quantification of many population genetic parameters, especially when the number of generated SNP loci is high (> 1,000 SNPs), individual sample size per population is low, or when patterns of population differentiation are especially subtle.

There are still trade-offs involved with using a RADseq based SNP approach versus a microsatellite approach, and there are times where a microsatellite approach may be all that is necessary to address the research questions of interest. The library preparation and high-throughput sequencing required by RADseq approaches tend to be more expensive and time-consuming than using microsatellite markers, and oftentimes require isolation of higher-quality DNA^112^. However, the cost-savings associated with using microsatellites are often limited to species where these markers have already undergone the relatively expensive sequencing needed to develop them. Other aspects of microsatellite marker application, including primer labeling, can also increase the cost of using this method. Furthermore, in order to accurately assess population genetic parameters using microsatellite markers, some studies have recommended as many as 25–30 individuals per sample population, which may offset any potential per-sample cost savings^113,114^. In contrast, simulation studies have shown that even with extremely low sample sizes (*n* = 2) genetic diversity and population genetic parameters can be accurately assessed using SNP markers as long as > 1,000 SNP loci are included in the analysis^115,116^. In studies where sample collection may be limited by logistical or biological constraints (e.g. low population densities, limited field time, or restrictive dive profiles), the extra time and financial investment to generate and analyze thousands of SNP loci may more efficiently and effectively identify ecologically relevant patterns of population genetic structure.

## Conclusions

This study used nine previously developed microsatellite loci and > 9,000 SNP loci generated using a 2bRAD approach to quantify the genetic structure of *M. cavernosa* across eight sites surrounding the Cuban archipelago. The complementary but differing results between microsatellite and SNP analyses for this sample set highlight some important trade-offs between these two approaches that researchers and MPA managers should consider when designing future coral population genetic studies. Sample collection accessibility and effort, sequencing costs, marker type and number, and relative desire to resolve subtle patterns in genetic differentiation are all considerations for method selection. For example, in this first investigation of *M. cavernosa* population genetics for Cuba, subtle but significant differentiation among sample populations was more clearly identified by the SNP approach. These results have important management implications on both local (within Cuba) and regional (throughout the TWA) scales. Coral reef management plans should consider local-scale connectivity dynamics when developing MPA regulations, possibly enforcing stricter policies in more highly connected coral populations to improve the efficacy of the MPA network as a whole. High levels of genetic connectivity for *M. cavernosa* have already been identified among highly distant reef populations (> 1,000 km separation between Belize and the Dry Tortugas)^7^ and perhaps Cuba may play an important role as a regional stepping stone among reefs in both the Gulf of Mexico and the western Caribbean. Future studies must obtain an understanding of Cuban coral populations’ role in regional metapopulation dynamics in order to effectively design networks of MPAs on an international scale for the mutual coral conservation benefit of multiple countries within this highly-connected system.

## Supplementary information

Supplementary file1

## Data Availability

Trimmed, de-duplicated, and quality-filtered 2bRAD sequences are uploaded to the National Center for Biotechnology Information Sequence Read Archive as part of BioProject PRJNA626681, accession numbers SAMN14647856 to SAMN14647942. Associated data files including microsatellite genotype data, STRUCTURE/NGSAdmix output files, analysis scripts, and protocols are available through the following GitHub repository: https://github.com/lexiebsturm/cubaMcavMsatSnp.
