# Supplementary material for: Population genetic structure of the great star coral, Montastraea cavernosa, across the Cuban archipelago with comparisons between microsatellite and SNP markers
Source: Sci Rep. 2020 Sep 22;10:15432. doi: 10.1038/s41598-020-72112-5 (PMC7508986; doi:10.1038/s41598-020-72112-5)
Supplement: Supplementary file 1 — Supplementary file1 [file 41598_2020_72112_MOESM1_ESM.pdf]

Supplementary information

Population genetic structure of the great star coral, *Montastraea cavernosa,* across the Cuban archipelago with comparisons between microsatellite and SNP markers

Sturm, Alexis B.[1*]; Eckert, Ryan J.[1]; González Méndez, Juliett[2,3]; González-Díaz, Patricia[3]; Voss, Joshua D.[1*]

[1] Florida Atlantic University, Harbor Branch Oceanographic Institute, 5600 N US Highway 1, Fort Pierce, FL, 34946, USA
[2] Centro Nacional de Áreas Protegidas, Calle 18a, No. 1441, Playa, La Habana, Cuba
[3] Centro de Investigaciones Marinas, Universidad de La Habana, Calle 16, No. 114, Miramar, La Habana, Cuba

*Correspondence: asturm2017@fau.edu, jvoss2@fau.edu

Supplementary Table S1. Observed (H_O) and Expected (H_e) heterozygosity values ± SE for each population generated from the microsatellite and SNP dataset.

	Microsatellite		SNP	
Population	Observed	Expected	Observed	Expected
Banco De San Antonio	0.389 ± 0.111	0.389 ± 0.057	0.204 ± 0.003	0.161 ± 0.002
Guanahacabibes	0.676 ± 0.053	0.708 ± 0.059	0.250 ± 0.002	0.256 ± 0.002
Isla De Juventud	0.704 ± 0.117	0.543 ± 0.090	0.248 ± 0.003	0.235 ± 0.002
Cayo Anclitas	0.733 ± 0.068	0.697 ± 0.059	0.249 ± 0.002	0.249 ± 0.002
Chivirico	0.677 ± 0.051	0.675 ± 0.058	0.254 ± 0.002	0.256 ± 0.002
Cabo Lucrecia	0.711 ± 0.068	0.704 ± 0.064	0.239 ± 0.002	0.239 ± 0.002
Cayo Sabinal	0.737 ± 0.054	0.723 ± 0.050	0.243 ± 0.002	0.246 ± 0.002
Cayo Jutías	0.639 ± 0.067	0.680 ± 0.066	0.228 ± 0.002	0.221 ± 0.002

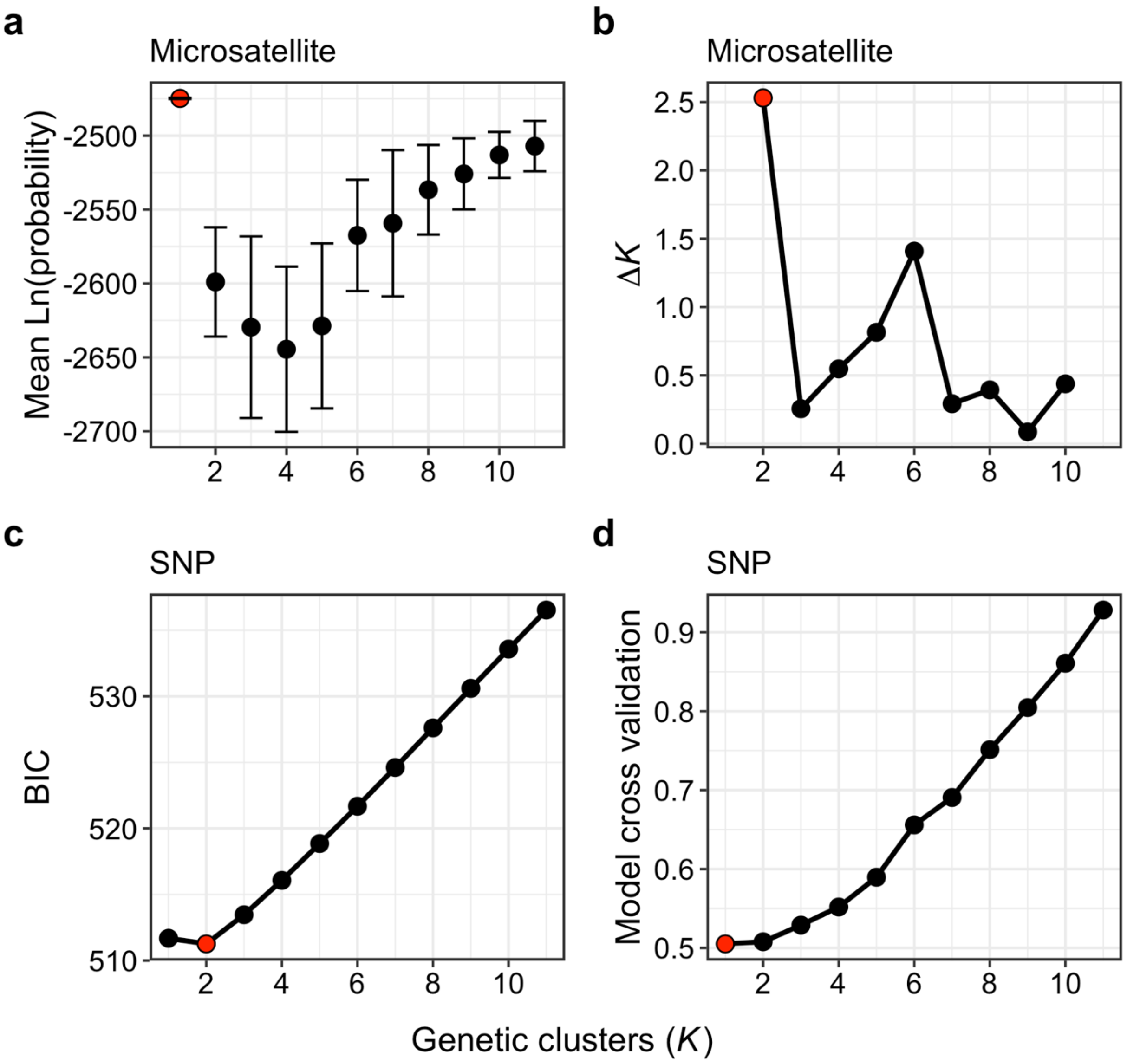

Supplementary Figure S1. Mean model simulations to determine the most likely value of genetic clusters (*K*, indicated by the red circle) using multiple population structure analysis methods for the (**a, b**) microsatellite and (**c, d**) SNP datasets. (**a**) $K = 1$ selection based on STRUCTURE log model likelihood (L(*K*)). (**b**) $K = 2$ selection based on STRUCTURE ΔK value generated using the Evanno method. (**c**) $K = 2$ selection based on Bayesian Information Criterion (BIC). (**d**) $K = 1$ selection based on ADMIXTURE model cross validation error.

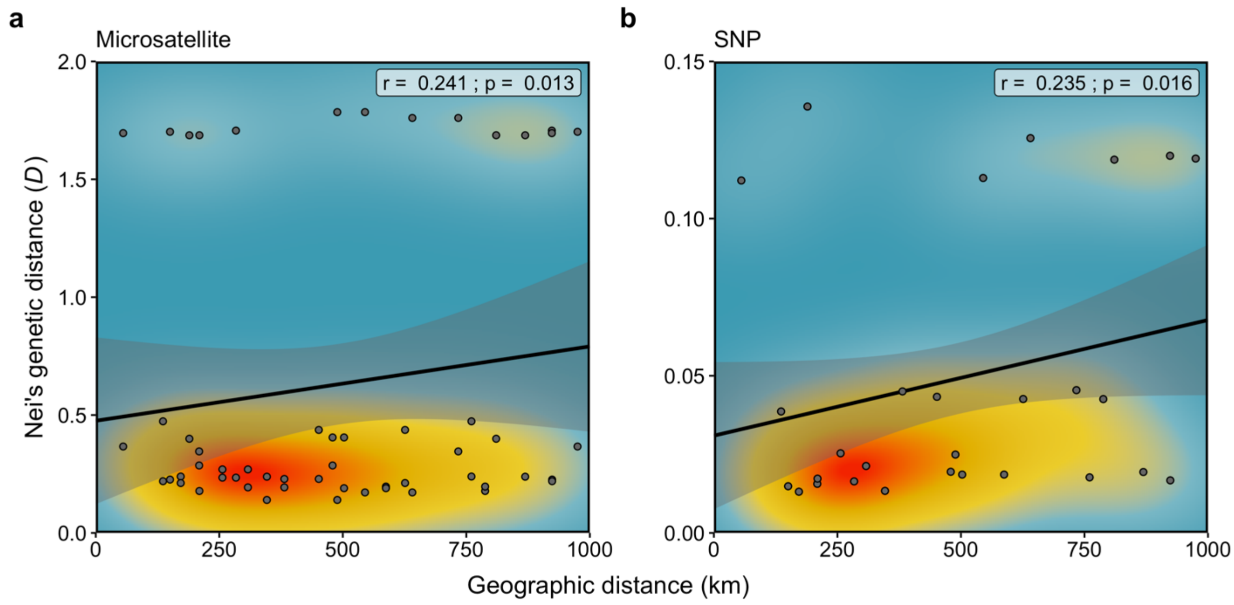

Supplementary Figure S2. Plots visualizing isolation-by-distance correlating Nei's genetic distance matrices and linear distance between sites (km) based on site GPS coordinates for (**a**) microsatellites and (**b**) SNPs (Mantel test, 999 permutations). Warm colors indicate increased density of pairwise genetic/geographic distance comparisons. Linear regression with 95% confidence interval and significant correlation coefficients are shown in each plot ($p < 0.05$).
